# Efficacy of Prophylactic Ketamine, Ondansetron, and Pethidine in Preventing Perioperative Shivering in Patients Undergoing Elective Knee Replacement Surgery Under Spinal Anaesthesia

**DOI:** 10.5152/TJAR.2021.20444

**Published:** 2022-02-01

**Authors:** Ramprasad Ramanathan, Rishabh Sethi, Shalendra Singh, Mudit Varshney, Debasish Das, Dheebakraj Nandagopalou, Deepak Dwivedi

**Affiliations:** 1 Department of Anaesthesiology & Critical Care, AICTS, Pune, India; 2 Department of Anaesthesiology & Critical Care, Army Hospital Research & Referral, New Delhi, India; 3 Department of Anaesthesiologist & Critical Care, Armed Forces Medical College, Pune, India

**Keywords:** Hypothermia, ketamine, ondansetron, pethidine sedation, shivering

## Abstract

**Objective:**

Perioperative shivering is a very common complication. Despite the vast array of knowledge regarding perioperative shivering and its after-effects, its prophylaxis is often overlooked. The study aims to compare the efficacy and safety of low-dose ketamine, ondansetron, and pethidine in the prevention of perioperative shivering in patients undergoing total knee replacement surgery under the subarachnoid block.

**Methods:**

In this randomized controlled study, 203 patients aged 18-75 were included and allocated to one of the 4 groups; normal saline (group S), ondansetron 4 mg (group O), ketamine 0.25 mg kg^−1^ (group K), and pethidine 0.25 mg kg^−1^ (group P). Side effects, namely hypotension, nausea and vomiting, sedation, hallucinations, and respiratory depression were recorded.

**Results:**

Perioperative shivering was present in 22 (44%), 8 (16%), 4 (7.84%), and 4 (7.69%) patients respectively in group S, O, K, and P, which was statistically significant when compared to group S with group K and P (*P *< .01). No difference in the incidence of hypothermia was observed across the groups (*P *< .17). A significantly lower incidence of hypotension was observed in group K. In group K, 5.9% of the patients were scored as being under severe sedation, according to the modified Wilson sedation scale. There was no incidence of hallucination or respiratory depression observed in any of the groups.

**Conclusions:**

Patients undergoing total knee replacement surgeries are highly predisposed to the development of hypothermia. Temperature monitoring is thus imperative for all patients. Prophylactic administration of low-dose ketamine or ondansetron or low-dose pethidine produces a significant anti-shivering effect without any significant side effects. However, low-dose ketamine has the advantages of a lower incidence of hypotension, nausea, and vomiting than pethidine.

## Main Points

- Despite the vast array of knowledge regarding perioperative shivering (POS) and its after-effects, its prophylaxis is often overlooked.
- Patients administered ketamine and pethidine showed the least POS.
- A significantly lower incidence of hypotension and severe sedation was observed in patients administered prophylactic ketamine.
- There was no incidence of hallucination or respiratory depression observed in any of the groups.

## Introduction

Perioperative shivering (POS) is a relatively frequent, unpleasant, and distressing experience, with varied incidence from 41-60% in patients under the subarachnoid block (SAB).^1,2^ It leads to frequent complications due to sympathetic stimulation and increased mortality and morbidity in the majority of elderly patients.^3,4^ Besides the interference with monitoring parameters, shivering may increase tissue oxygen consumption (by 100-600%) and cardiac output, and the circulating catecholamines lead to a rise in intracranial and intraocular pressure.^3,4^

One of the essential aspects of homeostasis is the maintenance of core body temperature. Post SAB, lower limb vasodilatation leads to redistribution of body heat from the central to the peripheral compartment. It abolishes behavioral mechanisms and disrupts the physiological mechanisms of thermoregulation, resulting in POS, a most distressing experience that is worse than surgical pain, as recalled by a few patients.^5^

Despite the vast array of knowledge regarding POS and its after-effects, its prophylaxis is often overlooked. A literature search revealed a large number of drugs for the prevention and treatment of POS, like ondansetron, pethidine, opioids, tramadol, nalbuphine, physostigmine, nefopam, and doxapram.^6-11^ Albeit the availability of many drugs, there is no consensus regarding the drug of choice. Our hospital, with a state-of-the-art Joint Replacement Centre (JRC), performs a high volume of total knee replacement (TKR) surgeries every day. The temperature in the JRC is maintained between 18°C and 20°C, against the conventional 22-24°C. In view of these prevalent conditions in the operating room, a higher incidence of POS is observed in patients undergoing SAB. This study was conducted to ferret out the drugs which would be a better choice for POS prophylaxis. The primary objective being evaluation and comparison of the relative efficacy and safety of low-dose ketamine, ondansetron, and pethidine in the prevention of POS in patients undergoing TKR surgery under SAB, the secondary objective was to study the side effects of these drugs.

## Methods

This study was carried out at the level I tertiary hospital. After approval from the Institutional Ethical Committee, written informed consent was obtained from the patients. A randomized control study with a drug and placebo, involving the American Society of Anaesthesiologists class I or II on a group of patients between 18 and 75 years of age undergoing TKR surgery under SAB, was included. Patients with known thyroid disease, Addison's disease, Parkinson's disease, Raynaud's syndrome, cardiopulmonary, liver, and kidney diseases, history of convulsions/epilepsy, bronchial asthma, and history of allergy to the agents to be used were excluded from the study. Patients requiring blood transfusion during surgery, or with initial axillary temperature <36.0°C or >37.5°C, use of sedative-hypnotic agents, vasodilators, antidepressant therapy with selective serotonin reuptake inhibitors and monoamine oxidase inhibitors, benzodiazepines, bleeding disorder, deformities of the spinal column, mental disturbance, or neurological diseases were also excluded. Patients having inadequate SAB requiring supplementation by general anaesthesia or contraindications to SAB were also excluded from the study. 

Before performing SAB, each patient was administered 10 mL kg^−1^ of ideal body weight (IBW) of lactated Ringer’s solution kept at room temperature and given without inline warming. Under strict aseptic conditions, SAB was administered at either the L2-3 or L3-4 interspaces with a volume of 2.0-3.0 mL of hyperbaric Bupivacaine, using a 25G Quincke spinal needle. Supplemental oxygen was delivered via a facemask throughout the procedure, starting immediately after administering SAB. According to a computer-generated randomization chart, the patients were assigned to one of the 4 groups. To ensure blinding, SAB was given by an anaesthesiologist who was not involved in the study. Each test drug was prepared blind in a 10 mL syringe and labeled “TEST DRUG” and administered by a nurse who was also blinded to group assignments, just after the SAB. Group S patients received an injection of normal saline 10 mL, group O patients received an injection of ondansetron 4 mg, group K patients received an injection of ketamine 0.25 mg kg^−1^, and group P patients received an injection of pethidine 0.25 mg kg^−1^. 

Patients were monitored with automated noninvasive blood pressure, pulse oximetry (SpO_2_), and electrocardiogram. Baseline blood pressure, respiratory rate, pulse rate, and SpO_2_ were recorded. Sensory block was assessed by the pinprick test. Body temperature was monitored with a temperature probe placed in the axilla and was recorded at 5-minute intervals. The patient was then moved to the operating room after the necessary preparation for knee replacement, 20-25 minutes after the SAB. The temperature in the main operating room was maintained at 18-22°C. All patients were covered with one layer of the standard blanket over the chest and abdomen during the operation. A surface warmer blowing warm air under the patient's blanket was also inducted. The incidence and severity of shivering were recorded at 5-minute intervals during the procedure. Shivering was graded using a scale similar to that validated by Tsai and Chu^12^ (0  =  no shivering, 1 =  piloerection or peripheral vasoconstriction but no visible shivering, 2  =  muscular activity in only one muscle group, 3  =  muscular activity in more than one muscle group but not generalized, and 4 =  shivering involving the whole body). If a score of 3 or more was obtained at any time, 15 minutes after SAB, the prophylaxis was regarded as ineffective, and an injection of tramadol 50 mg was then used in these patients as a salvage drug. Any score of 2 or more observed at any time, starting 15 minutes after SAB, was considered as positive for shivering. Sedation was considered based on the modified Wilson sedation scale (1 = oriented, eyes may be closed but can respond to “Can you tell me your name?” “Can you tell me where you are right now?”; 2 = drowsy, eyes may be closed, arousable only to command: “(name), please open your eyes”; 3 = arousable to mild physical stimulation (earlobe tug); 4 = unarousable to mild physical stimulation).^13^ Any score of 1 or more was considered positive for sedation. With the continuous axillary temperature monitoring, the temperature below 35.5°C any time during surgery was considered as hypothermia. Hypotension was considered as a decrease in the mean arterial pressure (MAP) of more than 20% from baseline. Severe hypotension was considered as a decrease of more than 25% in MAP or any reading of MAP < 65 mm Hg. Episodes of significant hypotension were treated with 6 mg mephentermine as intravenous (IV) bolus and then with further IV infusion of lactated Ringer's solution as required. Any episode of nausea or vomiting was recorded according to the patients' complaints, including complaints of retching. Respiratory depression was considered as a fall in SpO_2_ to less than 92% saturation or respiratory rate < 8 per minute. Side effects, namely hypotension, nausea and vomiting, sedation, hallucinations, and respiratory depression were duly recorded. Hallucination was measured using a validated hallucination screening instrument. If a patient’s response to any of the Questionnaire for Psychotic Experiences (QPE) screening questions suggested possible hallucinations, the full version of the QPE was planned to be administered as per the protocol.

## Statistical Analysis

Thus, considering a clinically significant reduction of shivering incidence from 40% to 10% in the intervention groups, we chose an alpha of 0.017 and a beta of 0.8, and calculated that 49 patients would be required in each study arm. Allowing for 10% loss to follow up, we aimed to recruit 54 patients in each group. Data analysis was carried out using STATA 9.0 (College Station, TX, USA). Data were presented as number (%) and mean ± SD/median (min-max) as appropriate. Baseline categorical variables were compared between the groups using the chi-square test/Fisher's exact test, and continuous variables were compared between the groups using one-way ANOVA. The primary and secondary outcomes were compared using Fisher's exact test. The value of *P* < .05 was considered as statistically significant.

## Results

**Figure 1.**
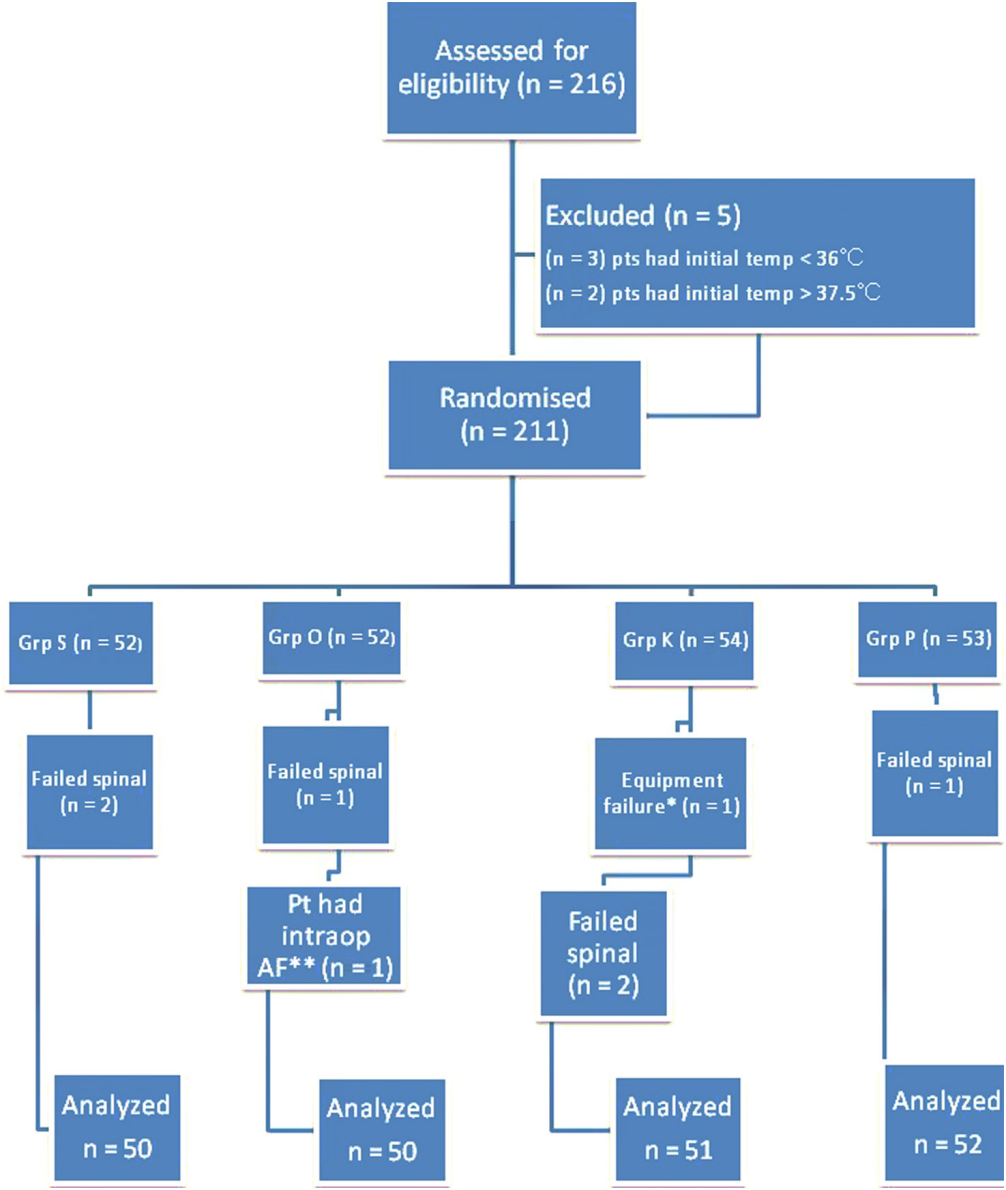
CONSORT diagram. *Temperature monitoring device not working; **Atrial Fibrillation.

The efficacy of prophylactic ketamine, ondansetron, and pethidine in preventing POS in patients undergoing elective TKR under SAB was studied in 203 patients. Demographic parameters and clinical characteristics were comparable between the groups (Table 1). Finally, 203 patients were analyzed in group S (n = 50), group O (n = 50), group K (n = 51), and group P (n = 52) (Figure 1).

The preloading volume of fluid infused ranged from 590 to 860 mL (10 mL kg-1 of IBW). No difference in the incidence of hypothermia was observed across the groups (*P *< .17) (Pearson’s *χ* ^2^ = 5.17, Pr = 0.15, Fisher's exact = 0.00) (Table 2). A significant decrease in POS was seen in group K (*P *< .01) (Table 2). Severe shivering was found the least in groups K and P (Table 2). In the intergroup comparison of group O with group K and with group P, the incidence of shivering, however, did not attain a statistically significant difference (*P* < .15, .14) (Pearson’s *χ* ^2^ =39.8, Pr = 0.0). 

A significantly lower incidence of hypotension was observed in group K (Pearson’s *χ* ^2^ = 24.09, Pr = 0.001, Fisher's exact = 0.17) (Table 3). No significant intergroup differences were observed in the incidence of bradycardia (*P* < .74) (Pearson’s *χ* ^2^ = 1.25, Pr = 0.74, Fisher's exact = 0.74) (Table 3). No incidence of nausea or vomiting was found in group O. No significant intergroup differences in drowsiness were observed between the groups (*P *< .23) (Table 3). In group K, 5.9% of the patients were found to have a score indicating severe sedation (Table 3).

The incidence of significant hypotension, i.e., those requiring mephentermine was also significantly lower in group K (Table 4). Further comparing the groups, a statistically insignificant difference in the incidence of hypotension was observed between control and group O and between control and group P (Table 4). The lower incidence of hypotension was significant in the comparison of group K with group P (*P *< .001) (Table 4). In a comparison of groups O and P, although both had a comparable incidence of hypotension overall, a significantly higher incidence of severe hypotension was observed in group P compared to group O (Table 4).

Leaving outpatients with failed spinal in each group which included those who either required any supplementation intraoperatively or required conversion to general anaesthesia, 1 patient in the group O who had an episode of Atrial Fibrillation (Hemodynamically stable) intraoperatively, and 1 patient in the group K in whom the initial temperature could not be obtained due to malfunctioning of the temperature probe. There was no incidence of hallucination noted in any of the groups. No incidence of respiratory depression was observed in any of the groups.

## Discussion

An important finding of our study was a significant reduction in the incidence of shivering in all the intervention groups. The literature revealed that the incidence of shivering has been reported to range from 41% to 60% when no prophylactic agent is used.^1,2,14^ As an outcome, the intervention groups recorded a much-reduced incidence of shivering, with 16% in group O, 7.84% in group K, and 7.69% in group P. Some studies that used 5-HT3 antagonists to attenuate shivering during SAB have observed a similar incidence. Noaman et al.^15^ compared ondansetron 4 mg with pethidine for prevention of shivering in SAB and found a similar anti-shivering effect and also recorded around 15% incidence of shivering in the ondansetron group. Nallam et al.^16^ used a higher dose of 8 mg ondansetron in their study on Indian patients for shivering prophylaxis in SAB for low-segment cesarean section and recorded a 10% incidence of shivering. Similar results were also observed by other authors.^17-19^ In our study, a smaller dose of 0.25 mg kg^−1^ of ketamine was used to minimize its side effects and get the best of potential benefits. The mechanism of prevention of hypotension by ketamine is the activation of the sympathetic nervous system, thereby often increasing blood pressure. In their study during general anaesthesia, Corredor et al.^20^ found shivering in 28% and 13% of the patients using ketamine 0.5 mg kg^−1^ and meperidine 0.4 mg kg^−1^ doses, but severe shivering was not reported in any patient given meperidine. Sagir et al.^17^ also used a similar prophylactic dose of 0.5 mg kg^−1^ IV ketamine, but in patients undergoing SAB, and found it to be effective in preventing POS. Using similar lower doses, Elmawgood et al.^21^ in their study in patients undergoing posterior vaginal repair surgeries under SAB found that the prophylactic administration of low-dose ketamine (0.25 mg kg^−1^) and hydrocortisone (2 mg kg^−1^) were comparable in reducing the incidence of shivering from 60% in the control group to 20% in the ketamine group.

We used pethidine as a study drug, considering it as a benchmark for the study, as it is one of the most established pharmacological agents used for the prevention of POS. In a study by Kelsaka E et al.^22^ pethidine 0.4 mg kg^−1^ IV was used as prophylaxis for POS. A reduction in the incidence of POS by up to 8% was observed in the pethidine group. 

The incidence of shivering across the groups in our study, however, showed no correlation to the incidence of hypothermia, as a comparable result was obtained for hypothermia incidence. The same has been observed by previous researchers; they found no concordance between axillary temperature and occurrence of shivering.^23^ This elaborates that the axillary temperature decreased significantly after SAB in all groups with respect to baseline values. 

A lesser incidence of hypothermia, although statistically insignificant, was observed in the ketamine group than in the other groups, which may be due to the vasoconstrictive effect of ketamine. This is consistent with previous studies, in which Sagir et al.^17^ observed that a lower incidence of decrease in core temperature was more in the ketamine (0.5 mg kg^−1^) group as compared to the control group. While pethidine disproportionately reduces the shivering threshold in comparison to its effect on the vasoconstriction threshold, ketamine interferes with thermoregulatory control mechanisms in the brain, thereby preventing shivering. Ondansetron also has a central mechanism of inhibition of the shivering response such that its anti-shivering effect is independent of the intraoperative core temperature, as observed by Powell and Buggy.^24^

A significantly lower incidence of hypotension was observed in the ketamine group (8%). The incidence of significant hypotension, i.e., requiring mephentermine, was also significantly lower in the ketamine group. Though most studies using ketamine with other drugs have reported a similar hemodynamic profile, a result similar to ours was observed in another study by Salah et al.^25^ which reported a significantly lower incidence of hypotension in the ketamine group in comparison with the control group. This has been attributed to ketamine having sympathetic stimulation and vasoconstrictive effects.

A significantly lower incidence of perioperative nausea/vomiting was observed in the ondansetron group and also in the ketamine group. The incidence of nausea and vomiting in the pethidine group was almost comparable to the control group in our study. Nausea and vomiting have been seen as important side effects of pethidine. However, by using a lower dose of pethidine (0.25 mg kg^−1^), we were able to minimize its incidence. Ondansetron is known to prevent nausea and vomiting, especially as it has powerful antiemetic effects. A similar result of decreased incidence of nausea and vomiting has been observed in previous studies. Ejiro et al.^26^ in their study of ondansetron versus tramadol in the prevention of POS following cesarean section under SAB reported a significantly lower incidence of nausea and vomiting in the ondansetron group using a dose of 4 mg.

Although a seemingly higher incidence of sedation was observed in the ketamine group, it was statistically insignificant. Sedation is a known effect seen with ketamine, but using a low dose of ketamine (0.25 mg kg^−1^), this effect was not very pronounced. In a previous study by Sagir et al.^17^ the use of 0.5 mg kg^−1^ of ketamine for shivering prophylaxis resulted in a high sedation (grade 3) in most patients in comparison to granisetron and control.

There are some limitations of the study. The scale we used to measure of shivering is the most commonly used shivering measurement tool referred to in literature. However, it may not be optimal for assessing POS occurring in association with SAB. Since under SAB, the lower body is unable to move, a scale 4 shivering intensity (entire body shivering) could never be observed. Probably another simplistic scale described by Crowley et al.^27^ could be better implied in such studies for patients undergoing SAB. The axillary temperature gives near-core and not actual core temperature readings, and may be less accurate and sensitive than the tympanic, nasopharyngeal, and esophageal temperatures. Preoperative volume status may affect intraoperative hypotension, so patients should be evaluated for their intravascular volume before surgery, using any measurement technique, e.g., the inferior vena cava diameter.

## Conclusion

Patients undergoing TKR surgeries under SAB are highly predisposed to the development of hypothermia. We conclude that prophylactic administration of low-dose ketamine or ondansetron or low-dose pethidine produces a significant anti-shivering effect without any significant side effects. However, low-dose ketamine has the advantages of a lower incidence of hypotension, nausea, and vomiting than pethidine.

## Figures and Tables

**Table 1.** Baseline Clinical Characteristics of the Patients in the Groups

Group	S	O	K	P	*P*
Age (mean ± SD)	64.96 ± 6.43	65.18 ± 5.06	65.05 ± 6.22	65 ± 5.70	.36
BMI (mean ± SD)	26.23 ± 3.83	26.4 ± 3.27	25.72 ±3.96	26.75 ± 4.3	.28
M/F	26/24	25/25	31/20	28/24	.69

M, male; F, female; BMI, body mass index; SD, standard deviation; K, ketamine; S, normal saline; O, ondansetron; P, pethidene.

**Table 2.** Shivering Scale in Different Groups of Patients

	Groups	Group S, n (%)	Group O, n (%)	Group K, n (%)	Group P, n (%)	*P*
Hypothermia		38 (76)	40 (80)	31(60.8)	37 (71.2)	.17
Shivering		22 (44)	8 (16)	4 (7.84)	4 (7.69)	.001 .002(S vs O) .001(S vs K) .001(S vs P) .15(O vs K) .14(O vs P .88(P vs K)
Shivering Scale	0	19 (38)	28 (56)	40 (78.4)	41 (78.3)	.001
	1	9 (18)	14 (28)	7 (13.7)	7 (13.5)	
	2	6 (12)	2 (4)	2 (3.9)	3 (5.8)	
3	16 (32)	6 (12)	2 (3.9)	1(1.9)

K, ketamine; S, normal saline; O, ondansetron; P, pethidine.

**Table 3.** Comparison of Patient Outcomes Between Groups

Patient Outcome		S, n (%)	O, n (%)	K, n (%)	P, n (%)	*P*
Hypotension		24 (48)	24 (48)	8 (15.7)	27 (51.9)	.001
Incidence of hypotension	No Hypotension	26 (52)	26 (52)	43 (84.3)	25 (48)	.001
	Moderate Hypotension	13 (26)	15(30)	8 (15.7)	21 (40.4)	
Severe hypotension	11(22)	9 (18)	0 (0)	6 (11.5)
Bradycardia		6 (12)	4 (8)	3 (5.6)	5 (9.6)	.74
Nausea/ vomiting reported		5 (10)	0 (0)	1 (2)	7 (13.5)	.009
Sedation		7 (14)	6 (12)	15 (29.4)	8 (15.4)	.23
Sedation score	1	43(86)	44 (88)	36 (70.6)	44 (84.6)	.23
	2	7 (14)	5 (10)	12 (23.5)	7 (13.5)	
3	0 (0)	1 (2)	3 (5.9)	1 (1.9)

K, ketamine; S, normal saline; O, ondansetron; P, pethidine.

**Table 4.** Incidence of Hypotension in Different Groups

		Group S (n = 50), n (%)	Group O (n = 50), n (%)	*P*
Hypotension	S versus O	24 (48)	24 (48)	1
Severe hypotension	11 (22)	9 (18)	.5
Hypotension	S versus K	24 (48)	8 (15.7)	.001
Severe hypotension	11 (22)	0 (0)	.001
Hypotension	O versus K	24 (48)	8 (15.7)	.001
Severe hypotension	9 (18)	0 (0)	.002
Hypotension	S versus P	24 (48)	27 (51.9)	.49
Severe hypotension	11(22)	6 (11.5)	.12
Hypotension	P versus K	27 (51.9)	8 (15.7)	.001
Severe hypotension	6 (11.5)	0 (0)	.014
Hypotension	O versus P	24 (48)	27 (51.9)	.29
Severe hypotension	9 (18)	6 (11.5)	.002

K, ketamine; S, normal saline; O, ondansetron; P, pethidine.
